# *PHGDH* is Key to a Prognostic Multigene Signature and a Potential Therapeutic Target in Acute Myeloid Leukemia

**DOI:** 10.7150/jca.90822

**Published:** 2024-03-11

**Authors:** Jiagui Zhong, Kezhi Huang, Shaofan Xie, Ailian Tan, Jiaqin Peng, Danian Nie, Liping Ma, Yiqing Li

**Affiliations:** 1 Department of Hematology, Sun Yat-Sen Memorial Hospital, Sun Yat-Sen University, Guangzhou 510120, China.; 2 Guangdong Provincial Key Laboratory of Malignant Tumor Epigenetics and Gene Regulation, Sun Yat-Sen Memorial Hospital, Sun Yat-Sen University, Guangzhou 510120, China.; 3 Department of Hematology, The Affiliated Kashi Hospital, Sun Yat-sen University, Kashi 844099, China.; 4 Internal Medicine Ward I, JieXi People's Hospital (Sun Yat-Sen Memorial Hospital, Sun Yat-Sen University-JieXi Medical Center), JieYang 515499, China

**Keywords:** Gene signature, Acute myeloid leukemia, Overall survival, *PHGDH*, Therapeutic target

## Abstract

As a rate-limiting enzyme for the serine biosynthesis pathway (SSP) in the initial step, phosphoglycerate dehydrogenase (*PHGDH*) is overexpressed in many different tumors, and pharmacological or genetic inhibition of *PHGDH* promotes antitumor effects. In the present research, by analyzing several acute myeloid leukemia (AML) datasets in the Gene Expression Omnibus (GEO), we identified prognosis-related genes and constructed a multigene signature by univariate, multivariate Cox regression and LASSO regression. Subsequently, the multigene signature was confirmed through Cox, Kaplan-Meier, and ROC analyses in the validation cohort. Moreover, *PHGDH* acted as a risk factor and was correlated with inferior overall survival. We further analysed other datasets and found that *PHGDH* was overexpressed in AML. Importantly, the expression of *PHGDH* was higher in drug-resistant AML compared to drug-sensitive ones. *In vitro* experiments showed that inhibition of *PHGDH* induced apoptosis and reduced proliferation in AML cells, and these antitumor effects could be related to the *Bcl-2*/*Bax* signaling pathway by the noncanonical or nonmetabolic functions of *PHGDH*. In summary, we constructed a twenty-gene signature that could predicate prognosis of AML patients and found that *PHGDH* may be a potential target for AML treatment.

## Introduction

Acute myeloid leukemia (AML) is a heterogeneous haematopoietic malignancy arising from the malignant clonal expansion of undifferentiated myeloid precursors with complex molecular pathogenesis, which includes a series of cytogenetic or chromosomal aberrations 1. Its pathological features are mainly manifested as the aberrant accumulation of undifferentiated myeloid progenitor cells in bone marrow with uncontrolled cell proliferation, obstruction of cell apoptosis, and arrested development. In addition, there is high heterogeneity both at the clinical level and prognosis: some patients might achieve long-term remission and survival, but others could become refractory or relapsed AML patients. These clinical heterogeneities of AML are related to several factors, of which age and cytogenetic and molecular alterations in tumour cells are particularly crucial 2. While such potential mutations may contribute to leukemic development and progression, they may be potential therapeutic targets that provide new insights for drug development 3.

Indeed, chromosomal aberrations and genetic mutations, including an increasing number of recurrent mutations, are considered drivers of AML and may significantly affect the prognosis of AML patients 4, 5. It has been suggested that, based on increasingly advanced sequencing technology, transcriptomic analysis can even provide predictive models with more prognostic value than traditional clinical parameters or genomic biomarkers 6. In recent years, many different studies have established survival-related multigene signatures 7-11. These gene signatures usually contain a cluster of genes, which may be of great significance for further study to discover critical genes from them.

In this study, we constructed a multigene signature containing twenty genes based on public databases, which was highly consistent with the survival of AML patients. Further analysis found phosphoglycerate dehydrogenase (*PHGDH*) was overexpressed in AML and correlated with inferior overall survival of AML patients. *In vitro* experiments confirmed that inhibition of *PHGDH* could effectively induce apoptosis and inhibit proliferation of AML cells, indicating that *PHGDH* may be a potential therapeutic target for AML treatment.

## Materials and methods

### Dataset acquisition

All original gene expression profile datasets (GSE37642, GSE9476, GSE106291) were downloaded from the Gene Expression Omnibus (GEO) database. The GSE37642 dataset includes GPL570, GPL96, and GPL97 platforms, of which the dataset based on the GPL570 platform contains 140 AML adult patients, and the other two platforms contain the same 422 AML adult patients. Here, we used only the data of the GPL570 and GPL96 platforms for analysis, with the data from the GPL96 platform as the training cohort and the GPL570 data as the validation cohort.

### Data screening and identification and validation of the prognostic gene signature

We filtered all data from the two platforms in the GSE37642 dataset, excluding the following data: 1. no survival status information; 2. overall survival (OS) less than 30 days; 3. uncertain diagnosis or not AML; 4. FAB classification (French-American-British system) unknown or M3 AML; 5. severe comorbidity; 6. presence of other malignancy; 7. prior anti-leukemic treatment. The inclusion criteria were:1. age starting from 18 years with no upper age limit; 2. newly diagnosed AML. First, in the training cohort, univariate Cox regression analysis was used to discover potential genes (p < 0.05) associated with survival. Then these genes were inputted into the LASSO Cox regression model analysis and the best penalty parameter lambda was tested using a 10-fold cross-validation 12, 13. With the optimal lambda value, the most important survival-related genes in newly diagnosed AML were detected. Next, prognosis-related risk profiles were established by stepwise multivariate Cox regression analysis. Each patient's risk score was calculated which was determined from the formula for the combination of the Cox coefficient and gene expression in this model 14. By using a cut-off point, the median risk score, patients in both cohorts were split into two groups: low- and high-risk. Kaplan‒Meier survival curves and logarithmic rank tests were used to assess survival differences between two groups. Then, we used the "survivalROC" package to compute the area under the receiver operating characteristic (ROC) curve (AUC). The "pheatmap" package was utilized to make a risk map in R. The same formula was also applied to verify the prognostic value of the multigene signature in the validation cohort. Finally, we performed separate survival analyses of these genes in the model in two cohorts to identify genes that had prognostic implications in both cohorts.

### Cell culture and patient samples

Human HL60, THP1, MV4‒11, and MOLM13 cells (all these four cell lines were purchased from ATCC) were cultured in RPMI 1640 medium (GIBCO, USA), with 10% foetal bovine serum and 100 units/ml penicillin and streptomycin and in a moistened environment of 5% CO_2_ at 37 °C. The *PHGDH* inhibitor NCT503 (S8619) was purchased from Selleck.

In this study, three healthy donors and three blood samples were acquired from newly diagnosed AML patients from our hospital in 2023. Before participation, written informed consent was obtained from all patients involved in this study. The research protocol was approved by the local ethics committee and conducted according to the ethical guidelines outlined by the World Medical Association Declaration of Helsinki.

### Cell viability assay

Cells were inoculated on 96-well tissue culture plates in triplicate at a density of 10,000 cells/wells and treated with different concentrations of NCT503 for 72 h. Each well was then added with 10 μL of Cell Counting Kit-8 solution assay reagent (CCK-8, APExBIO, USA), and the cultures were incubated at 37 °C for 2 to 3 h. Then a microplate reader (Biotek Synergy H1, USA) was used to measure the absorbance at 450 nm.

### Measurement of cell apoptosis

The Annexin V-FITC/PI Apoptosis Kit (E-CK-A211, Elabscience, China) was used to detect apoptotic cells by flow cytometry. After treatments, cells were collected and centrifuged for 5 min at 1,000 rpm and 4 °C. The supernatant was discarded and cells were resuspended in 150 μL mixture of 5 μL of annexin V-FITC and 145 μL 1× binding buffer, and incubated for 15 min at 37 ° in the dark. Then, 5 μL of propidium iodide (PI) solution and 145 μL 1× binding buffer were added for detection using flow cytometry. All experiments were repeated three times independently.

### Small interfering RNA (siRNA) and transient transfection

PHGDH siRNA was used to silence the PHGDH gene (PHGDH-si). A scrambled sequence siRNA (NC-si) was used as a negative control. The siRNA transfection was optimized using GP-transfectMate (Genepharma, Suzhou, China), according to the manufacturer's instructions.

### Western blotting

RIPA lysate (P0013, Beyotime Biotechnology, China) was used to extract the total protein of cells according to the instructions. The protein was separated by 10% SDS/PAGE and transferred onto a PVDF membrane (Millipore, Temecula, CA, USA). After blocking in 10% BSA solution for 1 h, the membranes were incubated with the primary antibody at 4 °C overnight. Subsequently, the membranes were incubated with secondary antibodies at room temperature for 2 h. Protein expression was detected with ECL reagents (G2020, Servicebio, China) and quantified by densitometry using ImageJ. All antibodies were anti-*PHGDH* (Proteintech, 14719-1-AP), anti-*Bcl-2* (Proteintech, 12789-1-AP), anti-*Bax* (Proteintech, 50599-2-Ig) and anti-β-actin (Proteintech,81115-1-RR).

### Statistical analysis

In this study, statistical analyses were performed by GraphPad Prism 9.0. Numerical data is displayed as the mean ± SD. All experiments and analyses were performed in triplicate. The data were assessed for statistical significance by one-way ANOVA and two-tailed Student's t-test according to the test. Statistical significance was defined as p <0.05 (*, p < 0.05; **, p < 0.01; and ***, p < 0.001).

## Results

The prognostic signature was constructed from the training cohort.

**Figure 1:**
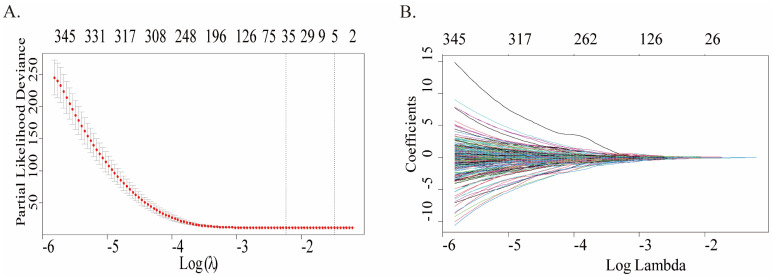
Construction of a prognostic multigene signature by LASSO regression analysis built on the training cohort. **(A)** Distribution of LASSO coefficient profiles of 35 genes. **(B)** Coefficient profile plot was created by using the log (lambda) sequence for selecting the best parameter (lambda).

A total of 422 cases were in the GPL96 platform of GSE37642, and only 348 cases met the requirements and were retained after screening and exclusion. Univariate Cox regression analysis was conducted to ascertain whether gene expression profiles were pertinent to overall survival (OS), and 2160 genes associated with survival prognosis were obtained. Fig.1A plots the coefficients for each gene, and the model showed the prognostic characteristics that were optimal when containing 35 genes (Fig.1B), which can be referred to in more detailed information (Table S1). To further screen for more meaningful genes, we used stepwise multiple Cox regression analysis to finally construct a predictive model composed of 20 genes, and their corresponding coefficients were shown in Table 1, of which 8 genes were risk factors and the remaining 12 were protective factors.

### Prognostic value of the multigene signature in the training and validation cohorts

**Figure 2:**
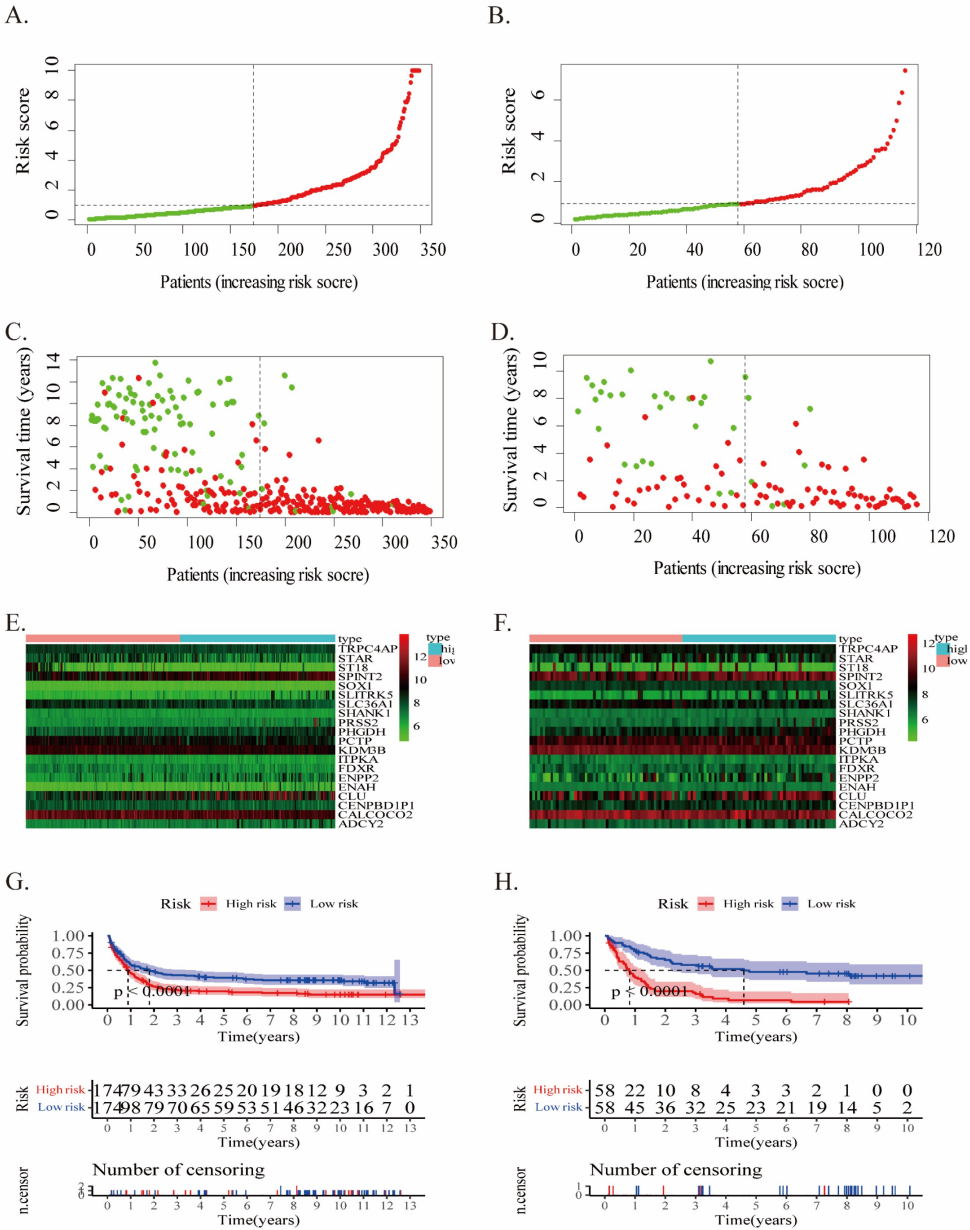
The twenty-gene signature's characteristics in the training (A/C/E/G) and validation (B/D/F/H) cohorts. **(A-B)** The distribution of the risk score (the red dot represents high risk). **(C-D)** patient survival time (the red dot represents death). **(E-F)** Expressions of twenty genes in high- and low-risk groups for the training **(G)** and validation **(H)** cohorts.

The risk score of each case in both cohorts was calculated using the gene expression levels together with their corresponding regression coefficients, resulting in a median risk score of 2.02. Accordingly, patients were split into two groups: low- (risk score <2.02) and high-risk (risk score ≥2.02). The prognostic value of risk score was assessed through evaluating survival differences in the low- and high-risk groups. Fig.2 displays the distribution of risk scores (Fig.2A, B), survival status (Fig.2C, D), and gene profiles (Fig.2E, F) for this twenty-gene signature in the two cohorts. The high-risk groups possessed more incidents and shorter OS than the low-risk groups. In fact, the heatmap showed that* SLITRK5*,* ENPP2*,* ADCY2*, *PRSS2*, and *PHGDH* were overexpressed in high-risk groups, but *ST18*, *STAR*, and *SLC36A1* were downregulated.

In the training cohort, Kaplan-Meier survival analysis displayed a poor prognosis trend for high-risk patients (p < 0.0001, Fig.2G). It was examined in the validation cohort in order to evaluate the effectiveness of this twenty-gene signature in predicting AML patients' OS (p < 0.0001, Fig.2H). The OS in the high-risk group was noticeably inferior to the low-risk group, along with previous results.

**Figure 3:**
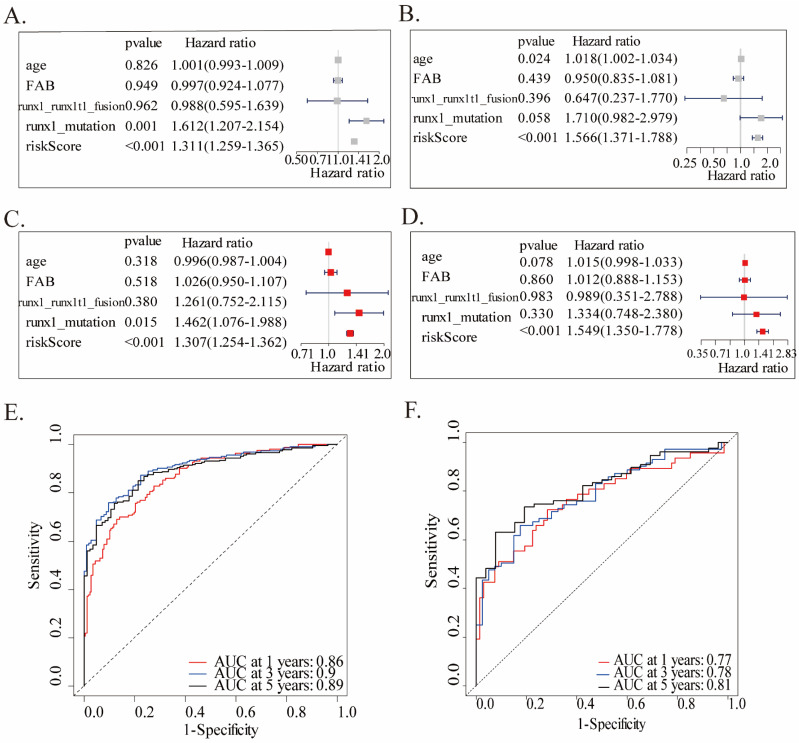
Univariate and multivariate Cox analyses and ROC analysis of the twenty-gene signature. Univariate and multivariate analyses are separately on the basis of this multigene signature and clinical covariates in the training **(A, C)** and validation **(B, D)** cohorts. ROC analysis of the overall survival prediction's sensitivity and specificity by 1-, 3-, and 5-year survival time in the training **(E)** and validation **(F)** cohorts. AUC stands for the area under the ROC curve.

We performed univariate and multivariate Cox analyses in both two cohorts, utilizing accessible variables provided in the dataset, including risk score, age, FAB classification, runx1_mutation, and runx1_runx1t1_fusion, to test the prognostic ability of this twenty-gene signature to clinical features. Both the two analyses of the training cohort found this twenty-gene signature to be a powerful predictor which was highly correlated with overall survival (HR = 1.311, 95% CI = 1.259-1.365, p < 0.001, Figures 3A; HR = 1.307, 95% CI = 1.254-1.362, p < 0.001, Fig.3C). Consistent with the training cohort, the signature showed a significant capability to predict OS in the validation cohort (Fig.3B, D). These findings confirmed that this twenty-gene signature was a powerful and independent variable. Moreover, ROC analysis was conducted to assess the prognostic accuracy of the twenty-gene signature model. Due to the large heterogeneity, rapid progression, and generally worse OS of AML patients, we tested the AUCs of 1-year, 3-year, and 5-year survival in the training cohort, which were 0.86, 0.90 and 0.89, reflecting a strong predictive power of this model (Fig.3E). Correspondingly, the validation cohort also confirmed this finding (AUC=0.77, 0.78, and 0.81, Fig.3F).

**Figure 4:**
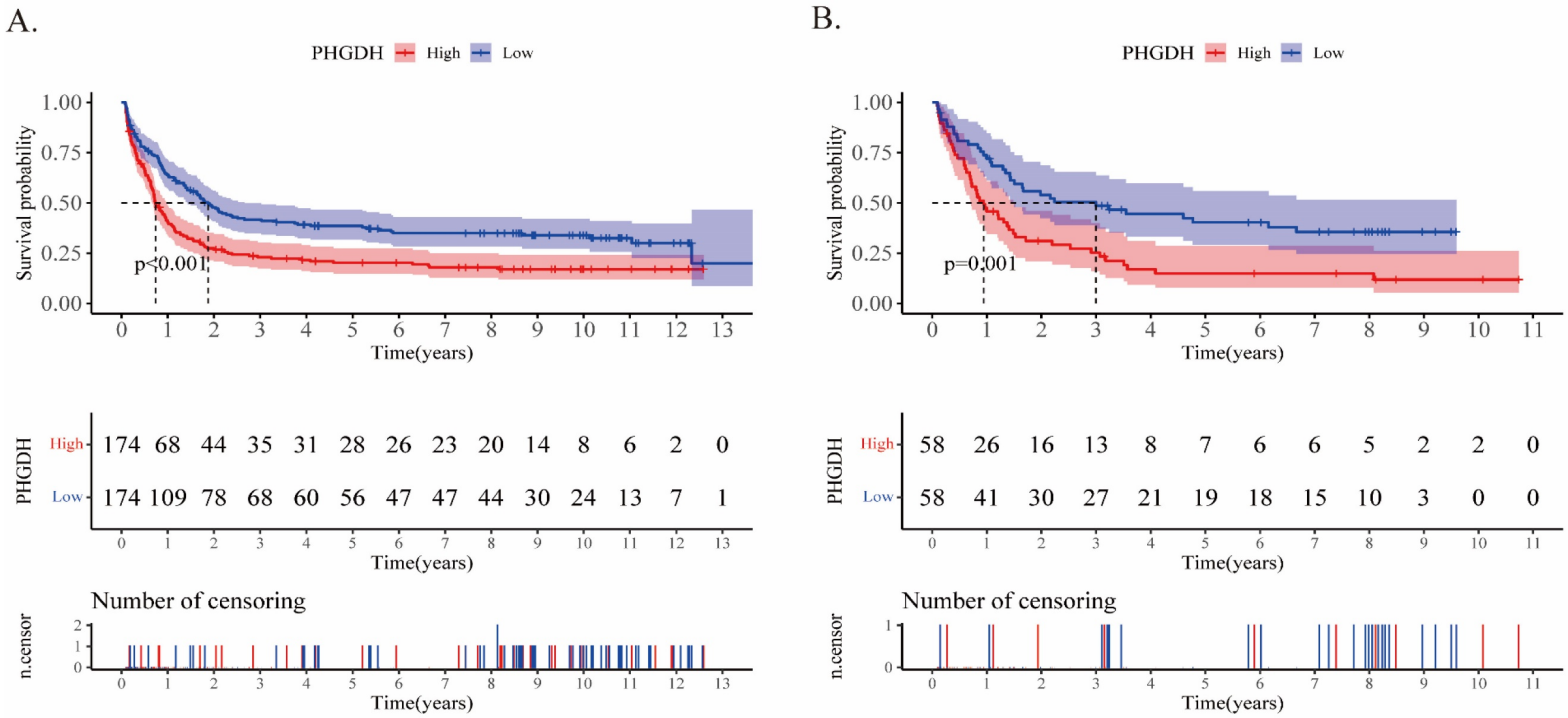
Kaplan‒Meier analyses of PHGDH in the training **(A)** and validation **(B)** cohorts. The two-sided log-rank test was used to identify differences between the two curves.

In addition, upon comparing the two heatmaps in Fig.2E and F, we found that 5 genes that overexpressed considerably in the high-risk patients of both cohorts included *SLITRK5*, *ENPP2*, *ADCY2*, *PRSS2*, and *PHGDH*. Furthermore, we conducted Kaplan‒Meier survival analysis for each of these twenty genes in the training and validation cohorts (Supplementary PDF document), and only *SLITRK5*, *PRSS2*, and *PHGDH* were associated with poorer overall survival in both cohorts (Fig.4). *SLITRK5*, a transmembrane protein named SLIT and NTRK-like protein-5, was a negative controller of hedgehog signaling in osteoblasts and a therapeutic target to promote bone formation 15. *PRSS2*, also known as serine protease 2, was reported to stimulate several solid tumor growth and progression 16, 17. Compared with these two genes, we are more interested in *PHGDH* and its role in AML. Therefore, we performed further analysis of *PHGDH*, one of the partially metabolized enzymes known to be dysregulated in cancer. *PHGDH* is the first rate-limiting enzyme that catalyzes serine synthesis, and its high expression activates the serine synthesis pathway (SSP) and thus promotes tumour growth 18. Since tumour cells have exceptional metabolic preferences to meet survival and proliferation needs, it may be a viable therapeutic strategy to treat tumours with *PHGDH* overexpression by targeting specific enzymes, such as *PHGDH* 19.

### *PHGDH* expression in AML

**Figure 5:**
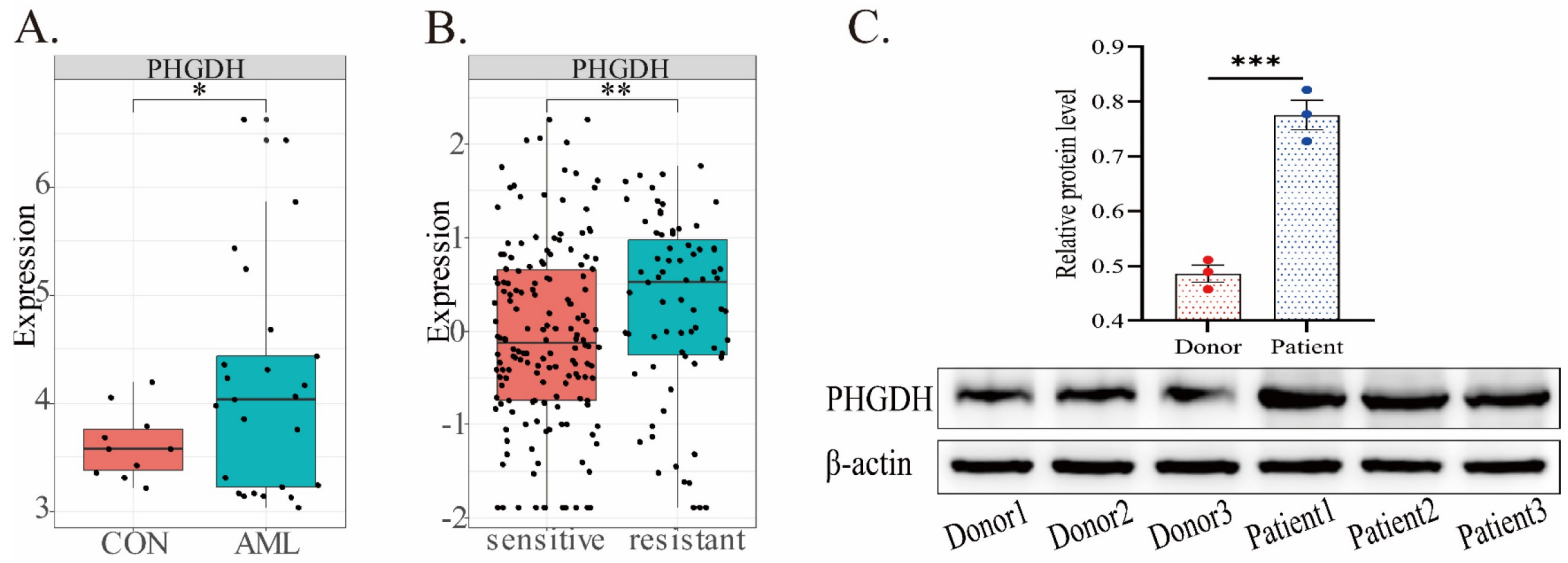
*PHGDH* expression in AML based on different public datasets. **(A)** In the GSE9476 dataset, the expression of PHGDH was considerably higher in AML patients than healthy individuals. **(B)** In the CSE106291 dataset, the drug-resistant group exhibited a higher level of PHGDH than the drug-sensitive group. **(C)** PHGDH protein expression levels in blood samples from 3 healthy donors and 3 AML patients. (*, *p* < 0.05; **, *p* < 0.01; ***, *p* < 0.001)

In the predictive model, *PHGDH* was discovered to be overexpressed in high-risk AML patients. Thus, we further compared the expression of *PHGDH* between healthy donors and AML patients, drug-sensitive and -resistant AML in public datasets (Fig.5). Our analysis of the GSE9476 dataset, comprising 38 healthy donors and 26 AML patients, revealed a significant upregulation of PHGDH expression in AML patients compared to healthy donors (Fig.5A). Similarly, analysis of blood samples from 3 healthy donors and 3 AML patients confirmed these findings by detecting the protein expression level of PHGDH(Fig.5C). The dataset CSE106291 has 235 patients, including drug-resistant and drug-sensitive patients, all of whom received induction therapy based on cytarabine and anthracycline (Fig.5B). In this dataset, we found that the *PHGDH* level was elevated in the drug-resistant group compared to the drug-sensitive group. These results imply that *PHGDH* may be a significant factor in AML and a potential therapeutic target.

### Effects of *PHGDH* inhibition in AML cells

**Figure 6:**
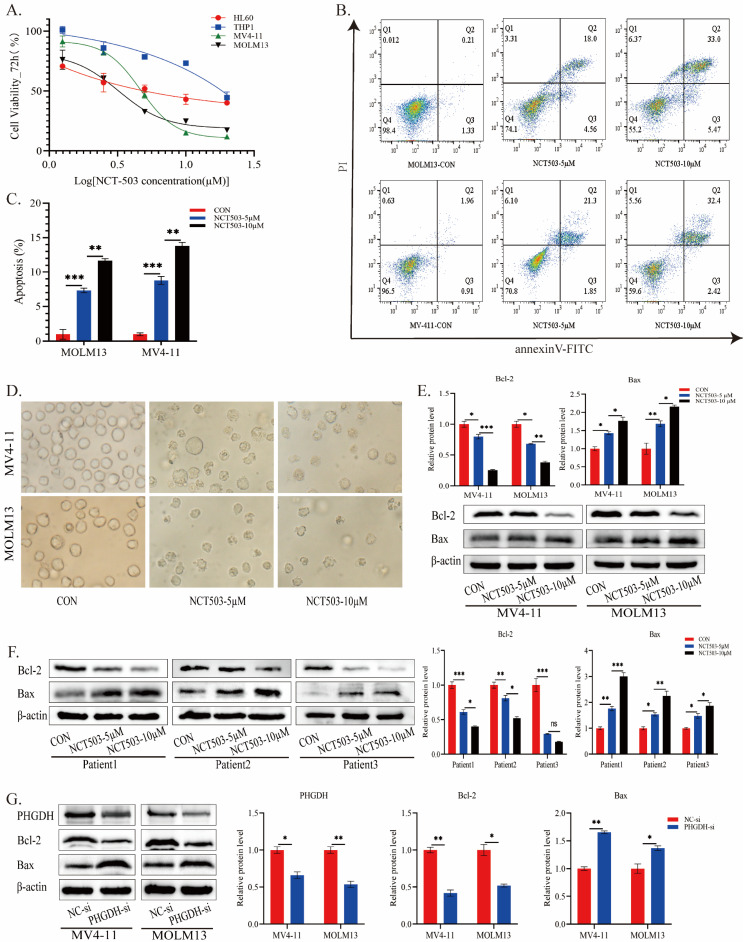
Effects of *PHGDH* inhibitor on growth inhibition and apoptosis induction in AML cells. **(A)** Cell viability after treatment with MOLM13 and MV-411 cells. **(B-C)** MOLM13 and MV-411 cells were treated with various concentrations of NCT-503 for 24 h. Subsequently, apoptosis levels were measured by flow cytometry. **(D)** Cell morphology changes after treatment with different concentrations of NCT503 for 24 hours(200X). The expression levels of *Bcl-2* and *Bax* in AML cell lines **(E)** and patient samples **(F)** were evaluated after treatment with NCT503 for 24 hours. **(G)** the protein levels of PHGDH, Bcl-2, and Bax were detected following PHGDH knockdown by PHGDH siRNA. Data of three independent experiments were presented as the mean ± standard deviation (SD). (*, *p* < 0.05; **, *p* < 0.01)

To further validate the effects of inhibiting *PHGDH* in AML, AML cell lines (HL60 and THP1 for FLT3-ITD^wt^; MV4-11 and MOLM13 for FLT3-ITD^+^) were treated with different concentrations of the *PHGDH* inhibitor NCT503. Fig.6A showed that pharmacological inhibition of *PHGDH* notably inhibited AML cell viability, and the effect was more pronounced in FLT3-ITD^+^ cells. Therefore, we used MV4-11 and MOLM13 cells for following experiments. The apoptosis test on FLT3-ITD^+^ cells found that NCT503 induced apoptosis in a concentration-dependent manner (Fig.6B-C). Previous studies confirmed that depletion of *PHGDH* could induce apoptosis by interacting with the anti-apoptotic protein *Bcl-2*, reducing the expression and stability of *Bcl-2*^20^-^22^. It remained unclear whether similar alterations occurred in FLT3-ITD^+^ AML cells when *PHGDH* was inhibited. Under microscopy, it was evident that cells treated with NCT503 exhibited diverse levels of fragmentation, amorphous shapes, and multi-directional morphology (Fig.6D). Consequently, we examined changes in the expression levels of antiapoptotic protein *Bcl-2* and proapoptotic protein *Bax*. The levels of *Bcl-2* was also attenuated in a dose-dependent manner, whereas *Bax* was upregulated in the same manner (Fig.6E). And similar changes have been observed in the cells of different patients with AML (Fig.6F). The results suggested that inhibiting *PHGDH* pharmacologically could inhibit the proliferation of FLT3-ITD+ AML cells and induce apoptosis and potentially involves activation of the *Bcl-2*/*Bax* signaling pathway.

To investigate the involvement of *PHGDH* in activating the *Bcl-2*/*Bax* signaling pathway, we used siRNA to knock down the protein level of *PHGDH* in AML cells (Fig.6G). Western blot analysis revealed that *PHGDH* knockdown resulted in up-regulation of the pro-apoptotic protein *Bax*, while the level of the anti-apoptotic protein *Bcl-2* was decreased, which was consistent with previous findings (Fig.6G). In summary, these findings enhanced our understanding of the potential therapeutic implications of targeting *PHGDH* in FLT3-ITD+ AML and the underlying mechanism may be related to the regulation of *Bcl-2*/*Bax* pathway by *PHGDH*.

## Discussion

Through comprehensive analysis of GEO databases, we built a twenty-gene signature for the prognostic characteristics of AML. By utilizing this signature in the training and validation cohorts, AML patients in low- and high-risk groups showed statistical significance in Cox regression models, Kaplan-Meier analysis, and ROC curves. All of these results demonstrated the efficiency and applicability of the twenty-gene signature in predicting the prognosis of AML.

Additionally, to identify genes that could be more important for prognostic influence among the twenty genes in the signature, we separately compared their differential expression in both cohorts and the impact on patient prognosis. We found an important gene *PHGDH* to be expressed higher in patients of the high-risk group than that in low-risk group, and patients with higher levels of *PHGDH* were related to inferior prognosis. We further verified that *PHGDH* was overexpressed in AML patients by public databases and patients' blood samples. Consistent with this, another study showed that *PHGDH* levels were significantly elevated in AML patients and that these patients tended to have a worse prognosis 9. These results suggest that *PHGDH* may be abnormally expressed in AML cells and be pivotal to cell metabolism.

*PHGDH*, a crucial metabolic enzyme of the de novo serine synthesis pathway (SSP), converts glycolysis- or gluconeogenesis-derived 3-PG into serine through a chain of enzymatic reactions together with other rate-limiting enzymes downstream of *PHGDH*, including phosphoserine aminotransferase 1 (*PSAT1*) and phosphoserine phosphatase (*PSPH*)^23^, ^24^. Subsequently, serine generates glycine through catalysis by serine hydroxy methyltransferases 1/2 (*SHMT1/2*). Both serine and glycine serve as raw materials for the synthesis of nucleotides, s-adenosylmethionine (SAM), and glutathione (GSH). While serine and glycine participate in nucleotide synthesis, enter one-carbon metabolism, and promote cancer cell proliferation, glutathione acts as a reactive oxygen species (ROS) scavenger to maintain redox balance in cells 25, 26. This is a highly regulated pathway in response to multiple metabolic stresses, including glucose and glutamine depletion, and the intermediate products NADH and GSH can regulate intracellular redox coordination 27.

In the course of tumour development, metabolic materials, such as amino acids and glucose, usually display aberrant alterations in their metabolic processes to meet the need for uncontrolled proliferation or other demands 28. One of the most important changes is the activation of SSP, thus increasing the synthesis of serine, which is utilized for protein and nucleotide synthesis, amino acid transport, and folate metabolism, regulating redox homeostasis and cell cycle progression, thus supporting tumour cell survival and proliferation 29-31. Many studies have confirmed the dysregulation of serine uptake or biosynthesis in tumour cells, as well as abnormal *PHGDH* expression, in different tumours, including lymphoma, multiple myeloma, glioma, hepatocellular carcinoma, melanoma, colon cancer, breast cancer, and others 32-38. These studies generally argued that intervention with *PHGDH* could affect tumour cell growth, but the underlying mechanism was not fully understood.

It is known that metabolic enzymes regulated by key signaling pathways in cancer cells can meet cellular metabolism and growth requirements by performing classical metabolic functions, but a growing number of studies have found that these metabolic enzymes can also support the rapid growth of cancer cells through noncanonical or nonmetabolic functions 39, 40. In addition to synthesizing serine, *PHGDH* may also promote tumour proliferation in noncanonical ways. Studies have shown that exogenous serine supplementation does not restore cell growth inhibition caused by *PHGDH* knockdown, suggesting that *PHGDH* may have important nonmetabolic functions in tumour development 35. *PHGDH* also interacts with the translation initiation factors eIF4E and eIF4A1 to promote translation initiation in the cytoplasm, thereby accelerating tumour development 41. *PHGDH* undergoes nuclear translocation after its phosphorylation, binds to c-Jun, and affects the transcription of target genes downstream of c-Jun related to cell growth regulation, thus promoting tumorigenesis 42. Similarly, *PHGDH* can promote growth and proliferation of hepatoma cells by activating mitochondrial translation and respiratory metabolism through noncanonical functions 43. In some other studies, *PHGDH* may perform noncanonical functions by the mitochondrial apoptotic pathway, facilitating the expression and stability of* Bcl-2* and eventually repressing cell apoptosis 20, 22. Our experiments also demonstrated that inhibition of *PHGDH* can induce apoptosis in AML cells, which may be related to the *Bcl-2/Bax* signaling pathway.

In addition, studies demonstrated *PHGDH* may be involved in cancer and tumour resistance to chemotherapy. Wei et al. found that *PHGDH* was a key driver of sorafenib resistance in hepatocellular carcinoma (HCC), and the synergistic effect of the *PHGDH* inhibitor NCT503 and sorafenib can effectively eliminate the growth of HCC *in vivo* 44. In multiple myeloma (MM), the level of *PHGDH* expression was considerably elevated and correlated with inferior survival, and the mechanism involved may be that high levels of *PHGDH* reduce ROS and DNA damage by increasing intracellular glutathione, promote the survival and proliferation of MM cells, and improve tumour cell resistance to bortezomib 45. Through a similar mechanism, *PHGDH* causes triple-negative breast cancer cells to become resistant to doxorubicin treatment 46. Based on the continuous discovery and improvement of *PHGDH* inhibitors, their effect in overcoming drug resistance or enhancing chemotherapy efficacy in tumour treatment may gradually become apparent.

These studies show that targeting *PHGDH* can not only directly intervene in tumour cell survival and proliferation through classical metabolic pathways or nonclassical pathways but also increase the sensitivity of cells to other drugs, indicating that it may be a promising therapeutic target in tumour treatment. To date, the antitumour effects of *PHGDH* in AML have been poorly studied. In recent years, researchers found that serine biosynthesis was a metabolic vulnerability of FLT3-ITD-driven AML. By inhibiting *PHGDH*, the proliferation of FLT3-ITD^+^ AML could be slowed 47. Our preliminary experiments also confirmed that inhibition of *PHGDH* significantly inhibited cell proliferation and induced apoptosis in AML cells with or without FLT3-ITD^+^ mutations. At the same dose, AML cells with FLT3-ITD^+^ mutations were inhibited to a greater extent. However, further research on its antitumour mechanism needs to be performed.

## Conclusions

In summary, our study constructed a multigene signature in AML. The signature was associated with AML OS and strongly identified the prognostic risk factors of AML patients. Remarkably, by analyzing twenty genes in the signature, we discovered a vital metabolism enzyme gene, *PHGDH*, which is overexpressed in AML patients and is related to inferior prognosis. In this study, we mainly investigated the impact of the noncanonical or nonmetabolic functions of *PHGDH* on AML. We preliminarily found that pharmacological inhibition of *PHGDH* can significantly inhibit cell proliferation and induce apoptosis in AML, which the *Bcl-2/Bax* signaling pathway might be involved in. *PHGDH* may play a crucial role in development of AML, making *PHGDH* a potential target for AML therapy.

## Supplementary Material

Supplementary methods, figure and tables.

## Figures and Tables

**Table 1:** Genes in the prognostic multigene signature. Stepwise multiple Cox regression analysis was used to construct the prognostic multigene signature composed of 20 genes, and their corresponding coefficients.

Gene symbol	Official Full name	Risk coefficient
*TRPC4AP*	Transient receptor potential cation channel subfamily C member 4 associated protein	-0.58191
*STAR*	Steroidogenic acute regulatory protein	-0.10973
*ST18*	ST18 C2H2C-type zinc finger transcription factor	-0.17558
*SPINT2*	Serine peptidase inhibitor, Kunitz type 2	0.13071
*SOX1*	SRY-box transcription factor 1	-0.95504
*SLITRK5*	SLIT and NTRK like family member 5	0.3104
*SLC36A1*	Solute carrier family 36 member 1	-0.33079
*SHANK1*	SH3 and multiple ankyrin repeat domains 1	-0.6686
*PRSS2*	serine protease 2	0.20974
*PHGDH*	Phosphoglycerate dehydrogenase	0.25727
*PCTP*	Phosphatidylcholine transfer protein	-0.24132
*KDM3B*	Lysine demethylase 3B	-0.47246
*ITPKA*	Inositol-trisphosphate 3-kinase A	-0.63094
*FDXR*	Ferredoxin reductase	-0.38926
*ENPP2*	Ectonucleotide pyrophosphatase/phosphodiesterase 2	0.14142
*ENAH*	ENAH actin regulator	0.22562
*CLU*	Clusterin	0.14254
*CENPBD1P1*	CENPB DNA-binding domains containing 2, pseudogene	-0.30834
*CALCOCO2*	Calcium binding and coiled-coil domain 2	-0.28088
*ADCY2*	Adenylate cyclase 2	0.25403
