# Supplementary material for: PHGDH is Key to a Prognostic Multigene Signature and a Potential Therapeutic Target in Acute Myeloid Leukemia
Source: J Cancer. 2024 Mar 11;15(9):2538–48. doi: 10.7150/jca.90822 (PMC10988303; doi:10.7150/jca.90822)
Supplement: Supplementary file 1 — Supplementary methods, figure and tables. [file jcav15p2538s1.pdf]

Supplementary Materials：

Supplementary methods

Reverse transcription-quantitative polymerase chain reaction (RT-qPCR)

Total RNA was extracted from the tissues and cells using TRIzol reagent (Sigma-Aldrich, Shanghai, China) and reverse transcribed to cDNA using cDNA synthesis kit (Vazyme, Nanjing, China). Gene expression was detected using the Roche LightCycler 96 system (Roche, Switzerland). The primer sequences used in this study were as follows: *PHGDH*, forward: 5'- CTGCGGAAAGTGCTCATCAGT -3', reverse: 5'- TGGCAGAGCGAACAATAAGGC -3'; *Bcl-2*, forward: 5'- GGTGGGGTCATGTGTGTGG -3', reverse: 5'- CGGTTCAGGTACTCAGTCATCC -3'; *Bax*, forward: 5'- CCCGAGAGGTCTTTTTCCGAG-3', reverse: 5'- CCAGCCCATGATGGTTCTGAT -3';β-actin forward: 5'- CAACCGCGAGAAGATGACC -3', reverse: 5'- ATCACGATGCCAGTGGTACG -3'. The relative expression level of genes was normalized to that of β-actin using the 2-ΔΔCt method.

Supplementary figure

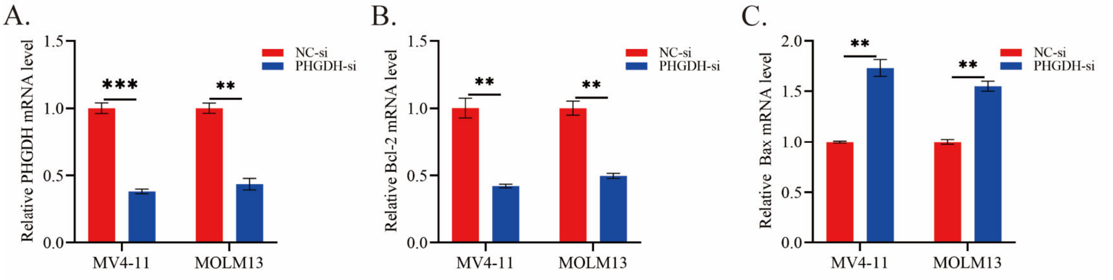

Fig. S1 The mRNA transcription levels of *PHGDH*(A), *Bcl2*(B), and *Bax*(C) were measured after *PHGDH* silence by *PHGDH* siRNA.

Supplementary table

Table S1. Identification of 35 survival-related genes using the LASSO Cox regression analysis at the optimal lambda value

Gene ID	Official Full Name	coefficient
ZNF415	zinc finger protein 415	0.011279
TRPC4AP	Transient receptor potential cation channel subfamily C member 4 associated protein	-0.12376
STAR	Steroidogenic acute regulatory protein	-0.00104
ST18	ST18 C2H2C-type zinc finger transcription factor	-0.0503
SPINT2	Serine peptidase inhibitor, Kunitz type 2	0.058017
SPAG1	Sperm associated antigen 1	0.000311
SOX1	SRY-box transcription factor 1	-0.12115
SOCS2	Suppressor of cytokine signaling 2	0.030876
SLITRK5	SLIT and NTRK like family member 5	0.096231
SLC36A1	Solute carrier family 36 member 1	-0.14538
SHANK1	SH3 and multiple ankyrin repeat domains 1	-0.17263
REC8	REC8 meiotic recombination protein	0.052116
PRSS2	Serine protease 2	0.034307
PHGDH	Phosphoglycerate dehydrogenase	0.156858
PCTP	Phosphatidylcholine transfer protein	-0.03168
NOV	Also known as CCN3,Cellular communication network factor 3	0.027493
LAPTM4B	Lysosomal protein transmembrane 4 beta	0.012866
KDM3B	Lysine demethylase 3B	-0.08249
KCTD15	Potassium channel tetramerization domain containing 15	-0.04894
ITPKA	Inositol-trisphosphate 3-kinase A	-0.17911
IL2RA	Interleukin 2 receptor subunit alpha	0.027723
HOPX	HOP homeobox	0.023423
FZD6	Frizzled class receptor 6	0.058051
FDXR	Ferredoxin reductase	-0.09727
ENPP2	Ectonucleotide pyrophosphatase/phosphodiesterase 2	0.004328
ENAH	ENAH actin regulator	0.035016
DNMT3B	DNA methyltransferase 3 beta	0.013679
DIAPH1	Diaphanous related formin 1	-0.02582
DDIT4	DNA damage inducible transcript 4	0.069492
COL6A1	Collagen type VI alpha 1 chain	0.023365
CLU	Clusterin	0.023515
CENPBD1P1	CENPB DNA-binding domains containing 2, pseudogene	-0.03018
CALCOCO2	Calcium binding and coiled-coil domain 2 [Homo sapiens	-0.13197
ADGRG1	Adhesion G protein-coupled receptor G1	0.011001

ADCY2	Adenylate cyclase 2	0.086271

Table S2. Association of PHGDH expression with clinical parameters of GSE37642_GPL96 348 samples

Clinical parameters	Total cases (%)	PHGDH Expression		
		High (%)	Low (%)	*P*
Age				
≥60	152(43.7)	77(50.7)	75(49.3)	0.286
<60	196(56.3)	88(44.9)	108(55.1)	
runx1-runx1t1_fusion				
No	327(94.0)	161(49.2)	166(50.8)	**0.011**
Yes	21(6.0)	4(19.0)	17(81.0)	
runx1_mutation				
No	294(84.5)	139(47.3)	155(52.7)	0.906
Yes	54(15.5)	26(48.1)	28(52.9)	
Overall Survival				
Dead	252(72.4)	132(52.4)	120(47.6)	0.003
Alive	96(27.6)	33(34.4)	63(65.6)	

*The two-tailed Pearson $\chi 2$ test or Fisher's exact test (when frequency < 5) was used to analyze the association of PHGDH expression and clinicopathologic parameters.

Table S3. Association of PHGDH expression with clinical parameters of GSE37642_GPL570 116 samples

Clinical parameters	Total cases (%)	PHGDH Expression		
		High (%)	Low (%)	*P*
Age				
≥60	57(49.1)	33(57.9)	24(42.1)	0.506
<60	59(50.9)	21(35.6)	38(64.4)	
runx1-runx1t1_fusion				
No	110(94.8)	54(49.1)	56(50.9)	**0.029**
Yes	6(5.2)	0(0)	6(100)	
runx1_mutation				
No	102(87.9)	46(45.1)	56(54.9)	0.004
Yes	14(12.1)	8(57.1)	6(42.9)	
Overall Survival				
Dead	83(71.6)	45(54.2)	38(45.8)	>0.999
Alive	33(29.4)	9(27.3)	24(72.7)	

*The two-tailed Pearson χ2 test or Fisher's exact test (when frequency < 5) was used to analyze the association of PHGDH expression and clinicopathologic parameters.

Table S4. Association of PHGDH expression with clinical parameters of GSE106291 235 samples

clinical parameters	Total cases (%)	PHGDH Expression		
		High (%)	Low (%)	*P*
Age				
≥60	113(48.1)	67(59.3)	46(40.7)	0.007
<60	122(51.9)	51(41.8)	71(58.2)	
Runx1-runx1t1_fusion				
No	229(97.4)	118(51.5)	111(49.5)	**0.014**
Yes	6(2.6)	0(0)	6(100)	
Runx1_mutation				
No	192(81.7)	92(47.9)	100(52.1)	0.137
Yes	43(18.3)	26(60.5)	17(39.5)	
Overall Survival				
Dead	135(57.4)	79(58.5)	56(41.5)	**0.003**
Alive	100(42.6)	39(39.0)	61(61.0)	
Drug response				
Resistant	71(30.2)	44(62.0)	27(38.0)	**0.018**
Sensitive	164(69.8)	74(45.1)	90(54.9)	

*The two-tailed Pearson $\chi 2$ test or Fisher's exact test (when frequency < 5) was used to analyze the association of PHGDH expression and clinicopathologic parameters.

Table S5. Association of PHGDH expression with clinicopathological features of GSE9476 26 AML samples

Clinicopathologic features	Total cases (%)	PHGDH Expression		
		**High (%)**	**Low (%)**	***P***
Age				
≥60	12(46.2)	6(50.0)	6(50.0)	>0.999
<60	14(53.8)	7(50.0)	7(50.0)	
FLT3 ITD				
No	13(56.5)	5(38.5)	8(61.5)	**0.414**
Yes	10(43.5)	6(60.0)	4(40.0)	
FLT3 TKD				
No	12(85.7)	6(50.0)	6(50.0)	>0.999
Yes	2(14.3)	1(50.0)	1(50.0)	
Gender				
Male	15(57.7)	8(53.3)	7(46.7)	**0.008**
Female	11(42.3)	5(45.5)	6(54.5)	

*The Fisher's exact test was used to analyze the association of PHGDH expression and clinicopathologic parameters (total sample is less than 40).

**Some groups had NA values and were excluded.

Table S6. Patient and healthy donor profile.

Number	Diagnosis	FLT3-ITD	Age	Gender
Patient 1	AML	low	45	male
Patient 2	AML	high	42	female
Patient 3	AML	high	52	male
Donor1	\	\	31	male

Donor2	\	\	49	male
Donor3	\	\	25	female

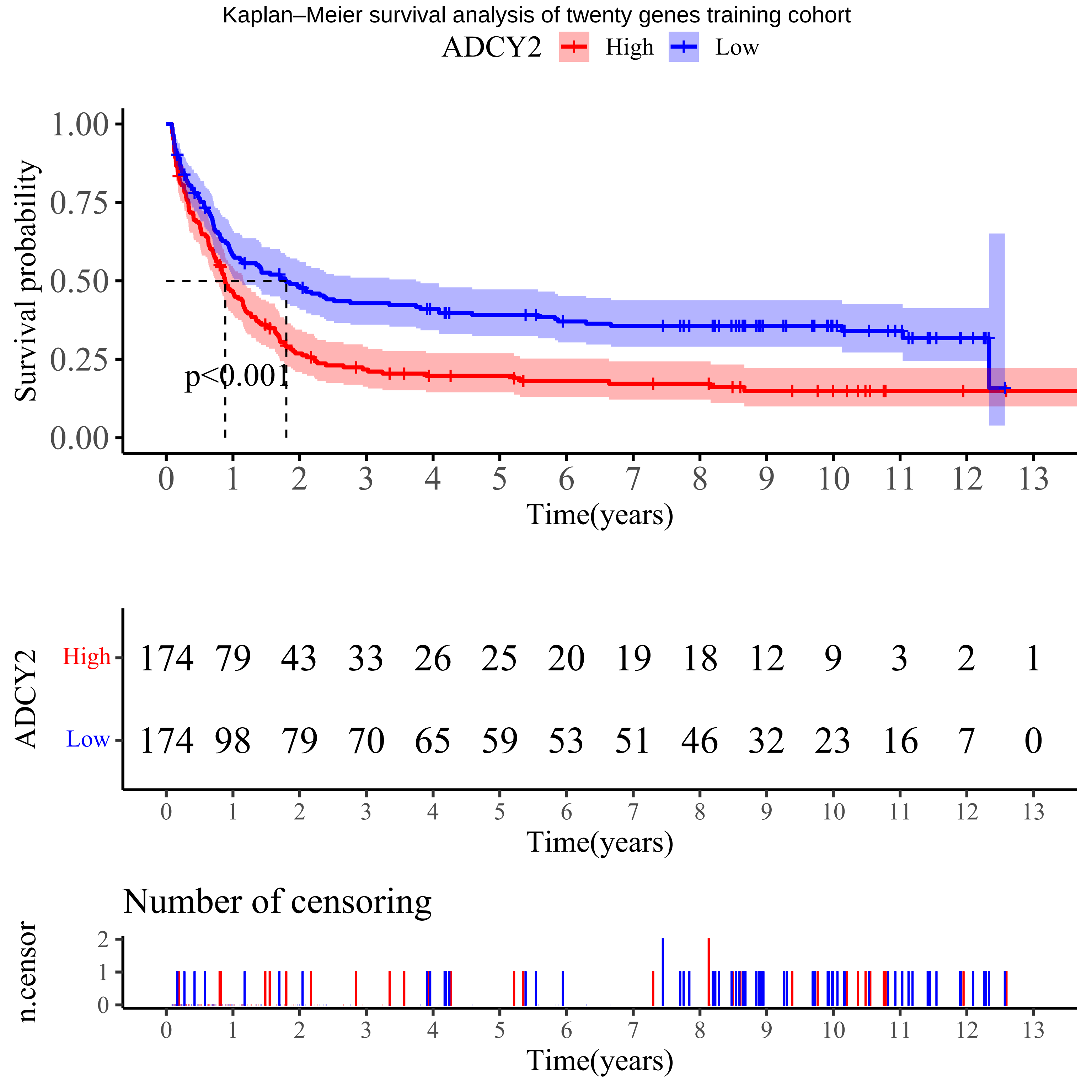

Kaplan–Meier survival analysis of twenty genes training cohort
ADCY2 High Low
Survival probability
1.00
0.75
0.50
0.25
0.00
p<0.001
0 1 2 3 4 5 6 7 8 9 10 11 12 13
Time(years)
ADCY2
High 174 79 43 33 26 25 20 19 18 12 9 3 2 1
Low 174 98 79 70 65 59 53 51 46 32 23 16 7 0
Time(years)
Number of censoring
n.censor
2
1
0
Time(years)
